# The Maternal Management of Children, in Health and Disease

**Published:** 1841-01

**Authors:** 


Art. VII. — *The Maternal Management of Children, in Health and Disease.* By Thomas Bull, m.d., Lecturer on Midwifery, &c.— *London*, 1840. 8vo, pp. 310.

Dr. Bull's work is written with nearly the same views and intentions as that of Dr. Combe, reviewed in our last Number. We did not, however, think it proper to place them together in the same article, as it would have been unjust to Dr. Bull to subject him to a close comparison with so very philosophical and so very superior a writer as Dr. Combe. Dr. Bull's work, nevertheless, is one of fair average merit, and contains many useful directions for mothers as to the best mode of managing children in health and disease.

